# Knockout of Formyl Peptide Receptor-1 Attenuates Cigarette Smoke–Induced Airway Inflammation in Mice

**DOI:** 10.3389/fphar.2021.632225

**Published:** 2021-04-26

**Authors:** Lijuan Gao, Ni Zeng, Zhicheng Yuan, Tao Wang, Lei Chen, Deqing Yang, Dan Xu, Chun Wan, Fuqiang Wen, Yongchun Shen

**Affiliations:** Department of Respiratory and Critical Care Medicine, West China Hospital of Sichuan University and Division of Pulmonary Diseases, State Key Laboratory of Biotherapy of China, Chengdu, China

**Keywords:** formyl peptide receptor-1, cigarette smoke, airway inflammation, bioinformatics analysis, NF-κB

## Abstract

**Objective: **The formyl peptide receptor-1 (FPR-1) has been reported to be implicated in the regulation of inflammatory disorders, while its role in cigarette smoke (CS)–induced airway inflammation has not been fully explained. In this study, we investigated the role of FPR-1 in CS-induced airway inflammation and the possible mechanism through gene knockout (KO) technology and transcriptional study.

**Methods: **FPR-1 KO or wild-type C57BL/6 mice were exposed to mainstream CS to establish an airway inflammation model. Cell counts and pro-inflammatory cytokines were measured in bronchoalveolar lavage fluid (BALF). Lung tissues were collected for histological examination, polymerase chain reaction, Western blot, transcriptomic gene study, and related bioinformatics analysis.

**Results: **CS exposure induced significant histological inflammatory changes, increased neutrophils, and pro-inflammatory cytokines in the BALF of wild-type mice, which were all attenuated by KO of FPR-1. The transcriptomic gene study showed a total of 198 up-regulated genes and 282 down-regulated genes in mouse lungs. Bioinformatics analysis including Gene Ontology (GO) and Kyoto Encyclopedia of Genes and Genomes (KEGG) suggested these differentiated expressed genes were significantly related to the immune, chemotaxis responses, and cross-talked with a complicated network of signaling pathways including NF-κB. Western blot validated that KO of FPR-1 inhibited CS-induced NF-κB activation.

**Conclusion: **Knockout of FPR-1 significantly ameliorates CS-induced airway inflammation in mice, possibly via its related immune-chemotaxis responses and inhibition of NF-κB activation.

## Introduction

Chronic obstructive pulmonary disease (COPD) is characterized by persistent respiratory symptoms, airflow limitation, and chronic airway inflammation (Wang et al., 2018), and it was estimated that there are 99.9 million people with COPD in China based on the latest epidemiological studies (Wang et al., 2018). COPD is a major cause of chronic morbidity and mortality throughout the world, and COPD was the third leading cause of death in the United States in 2011 (Hoyert and Xu, 2012).Thus, COPD places a heavy burden on public health. The pathogenesis of COPD is so complicated, but it is well recognized that cigarette smoke (CS) is the main risk factor of COPD. CS contains thousands of toxics that cause activation of inflammatory cells, injuries of airway epithelial cells, and CS-induced airway inflammation is the key feature of COPD (Caramori et al., 2015; Aghapour et al., 2018). To protect CS-induced airway inflammation is of great importance for the management of COPD.

Growing studies suggested that the CS-induced imbalances between protease and antiprotease, oxidant and antioxidant were the basis of pathophysiology in COPD. It has demonstrated that neutrophil elastase, neutrophil chemotaxis, oxidant production, and cytokine release of neutrophil were involved in the pathogenesis of COPD (Dunne et al., 2019; Fessler, 2019). Meanwhile, severe decrease in the number and function of peripheral T cells did not influence the development of pulmonary changes induced by CS in mice (De Cunto et al., 2016) and lung inflammation still persisted after smoking cessation in mice (De Cunto et al., 2018). In general, it suggested that innate immunity is a leading actor in the early and late development of pulmonary changes in COPD. Recent studies suggest that formyl peptide receptors (FPRs) have been shown to function in different innate immune cells, including regulating the neutrophil migration, stimulating inflammatory cytokine release from monocytes, and regulating the maturation and migration of dendritic cells (Banoth and Cassel, 2018). The formyl peptide receptor-1 (FPR-1) is one member of the FPRs family and is potentially a key receptor within the inflammatory process (Dorward et al., 2015; Yang and Hwang, 2016). FPR-1 knocks out and FPR-1 antagonist attenuated this inflammation process of acute lung injury; FPR-1 may be a novel therapeutic target for acute respiratory distress syndrome through the regulation of inflammation (Dorward et al., 2017). Our team also reported that FPR-1 was involved in pro-inflammatory responses induced by mitochondrial peptides in alveolar epithelium, thus, played a potential role in acute lung injury (Zhang et al., 2018). Besides, studies demonstrated that formyl peptides were present in tobacco leaves, secreted by pathogens or released from dying host cells after tissue injury, and their receptor FPRs were involved in the initiation and progression of inflammation in COPD (De Cunto et al., 2020a). Moreover, FPR-1 has been reported to play a role in CS-induced lung emphysema in mice (Cardini et al., 2012), ongoing lung inflammation, and progression of COPD in mice (De Cunto et al., 2018). These studies confirmed the role of FPR-1 in the regulation of inflammation, and therapy targeted on FPR-1 may provide a novel direction for inflammatory lung diseases. Although the association between FPR-1 and smoking-induced lung emphysema in mice has been explored, the mechanism under FPR-1 modulation has not been fully examined. And the current study aimed to investigate the effects and molecular mechanisms of FPR-1 in CS-induced airway inflammation through the gene knockout (KO) animal study, transcriptomic study, and followed by bioinformatics analysis.

## Materials and Methods

### Animal Preparation

The FPR-1 KO mice on a C57BL/6 background were generated via CRISPR/Cas9 gene targeting technology by Cyagen Biosciences (Suzhou, China). Four groups of 8–10-weeks-old, male C57BL/6 mice, wild-type (WT), WT exposed to CS (WT + CS), FPR-1 KO mice (KO), and FPR-1 KO mice exposed to CS (KO + CS) (*n* = 6 in each group) were housed in a specific pathogen-free and a temperature-controlled (22°C) room with a 12:12 h light–dark cycle. The mice were free to food and water. This study was approved by the Panel on Laboratory Animal Care of West China Hospital of Sichuan University.

### Smoke Exposure

Mice were allowed to adjust to the animal housing facilities for one week before any interventions were carried out. For cigarette smoke exposure experiment, the WT + CS and KO + CS groups were exposed to Marlboro cigarettes (Marlboro^®^, Philips Morris, United States, with 1.1 mg nicotine and 11 mg tar per cigarette) with the standard method used in our group (Aghapour et al., 2018), with particulate matter concentration in the chamber at about 300 mg/m^3^; briefly, the mouse was restricted in a custom-designed nose-only exposure tube (China Pattern number: 201810,963,763.4), in which the nose of the mouse was exposed to the smoke or air through a one-way flow opening in front of the nosepiece. The restrained mice were then mounted to a smoking chamber constructed with a flow-in layer in the middle and the flow-out layers at two sides for 75 min twice daily, 5 days per week for up to 4 weeks using a Baumgartner-Jaeger CSM 2082i automated cigarette smoking machine (CH Technologies, West-Wood, NJ, United States). The other two groups were exposed to filtered air following the same schedule.

### Bronchoalveolar Lavage Fluid Cell Counting

After four weeks, mice were intraperitoneally anesthetized with 50 mg/kg sodium pentobarbital, followed by which the left lung was lavaged 3 times with 0.3 ml volume of ice-cold phosphate buffer saline (PBS), with a recovery rate of 80%, and the recovered bronchoalveolar lavage fluid (BALF) was centrifuged at 1,000 g at 4 °C for 5 min. The upper fluid samples were immediately frozen at −80 °C for further measurement. The BALF cell counting was performed by the standard method which was described in our previous work (Chen et al., 2017). The total cell number was determined by a hemocytometer, and differential cell count (neutrophils, macrophages, and lymphocytes) was performed by cytocentrifugation (Cytopro7620, Wescor, Utah, United States) at 700 rpm for 10 min and stained with Wright’s stain (200 cells were counted for each mouse). The cells counting were finished by two independent experienced investigators, who were blinded to the experimental conditions.

### Inflammatory Cytokine Measurement

The levels of these cytokines interleukin (IL)-1β, IL-6, and tumor necrosis factor (TNF)-α in BALF were measured using enzyme-linked immunosorbent assay (ELISA) kits (Develop, China) following the manufacturer's instructions to assess the inflammation level. The cytokine measurement was finished by independent experienced investigators, who were blinded to the experimental conditions.

### Lung Histology Examination

The right middle lobe was fixed in 4% paraformaldehyde (pH 7.4) overnight and embedded in paraffin and cut into 4-mm-thick sections. The paraffin sections were then stained with hematoxylin and eosin (HE) to evaluate the morphological changes and inflammation in the lungs. Histologic inflammatory scores were obtained by an experienced histologist blinded to experimental details, according to the evaluation of perivascular infiltration, peribronchial infiltration, parenchymal infiltration, and epithelial damage. For each evaluation index, 0 point represents no sign of disease whereas five point indicates serve inflammation, thus the inflammation degree was presented as a total score point between 0 and 20 (Blanchet et al., 2005).

### Transcriptomic Studies

In this part of study, mRNA isolation, cDNA library construction, and sequencing were performed by the Oebiotech Co. Ltd. (Shanghai, China). Briefly, total RNA was extracted from each lung tissue using the Trizol reagent (Life Technologies) and digested with DNase and further purified using the mini-elute kit (Qiagen) according to the manufacturer’s protocol. The integrity of the RNA was evaluated on an Agilent Bioanalyzer, and samples with RNA integrity numbers >7.0 were prepared for sequencing. Next, Poly(A)-tailed RNA was prepared by using the mRNA Seq Sample Prep Kit (Illumina) and used to create libraries for the deep sequencing studies, and samples were sequenced on Illumina Hiseq 4000 by using a PE150 model.

Reads from every sample were mapped to the genome using TopHat version2.1.1, and we obtained an alignment of the trimmed reads to the reference genome in the BAM format. Aligned reads were visualized using a local copy of the Integrative Genomics Viewer (www.broadinstitute.org/igv/). The output files generated from hisat2 were converted into files viewable in IGV by BEDTools and then further processed by the “count” function in igvtools (included with the IGV software) to create an average alignment track viewable as a bar chart. The log2 of the frequency of the reads was plotted to better visualize the extensive range of the read coverage. Raw transcriptomic data were uploaded in the China National Center for Bioinformation—National Genomics Data Center (accession: CRA003586).

### Bioinformatics Analysis

Gene expression was reported in Fragments Per Kilobase of exon per Million fragments mapped (FPKM). Differentially expressed genes (DEGs) were analyzed using DESeq R packages according to the package’s manual. Genes that at least 1-fold upregulated or downregulated and with *p* value <0.05 were considered as DEGs. The resulting gene list was then annotated according to the Gene Ontology (GO) and Kyoto Encyclopedia of Genes and Genomes (KEGG) databases with the R package named clusterProfiler based on the hyper-geometric distribution (Huang da et al., 2009). Then, the significantly enriched GO or KEGG terms were analyzed using the hyper geometric test with a *p* value ≤ 0.05.

### Real-Time qPCR

To further validate the sequencing results, real-time qPCR was performed for the selected genes. RNA was converted to cDNA with PrimeScript RT reagent Kit with gDNA Eraser (Takara, Japan) according to the manufacturer's protocol. Real-time qPCR analysis was conducted in triplicates by the LightCycler 96 real-time PCR detection system using Faststart Essential DNA Green Master (Roche, Switzerland). The primer sequences were summarized in Supplementary Table S1. All data were normalized to *β*-actin gene expression, and relative expression levels were determined using the 2^−ΔΔCt^ method.

### Protein Extraction and Western Blotting

Lung tissues were collected, and total proteins were extracted using lysis buffer containing RIPA (Beyotime Biotechnology, China), protein phosphatase inhibitor cocktail (Applygen Technologies, China), and PMSF (Beyotime Biotechnology, China). Whole lysates were collected and centrifuged at 12,000 rpm at 4°C for 20 min. Protein concentrations were detected using BCA Protein Assay Kit (Thermo Fisher Scientific, United States). Then, the lysates were loaded on sodium dodecyl SDS-PAGE with 10% running gel and transferred onto PVDF membranes (Millipore, United States). 5% BSA was used to block the membranes for 2 h. Then, the membranes were incubated with primary antibodies to NF-κB P65 (1:1,000; Cell Signaling Technology, United States), phospho- NF-κB P65 (1:1,000; Cell Signaling Technology, United States), IκBα (1:1,000; Cell Signaling Technology, United States), and *β*-actin (1:3,000; Cell Signaling Technology, United States) overnight at 4°C and washed with TBST three times for 5 min each, followed by incubation in a secondary antibody for 2 h. After being washed with TBST, the loaded proteins were visualized by enhanced chemiluminescence reagents. The density of the protein bands was quantified using ImageJ imaging software (Media Cybernetics, Rockville, MD, United States).

### Statistical Analysis

The data were assessed as the mean ± SEM. A statistical analysis was performed using a one-way analysis of variance (ANOVA) for studies with more than two groups. Differences were considered statistically significant if *p* < 0.05.

## Results

### CS-Induced Airway Inflammation Was Diminished in FPR-1 Knockout Mice

**FIGURE 1:**
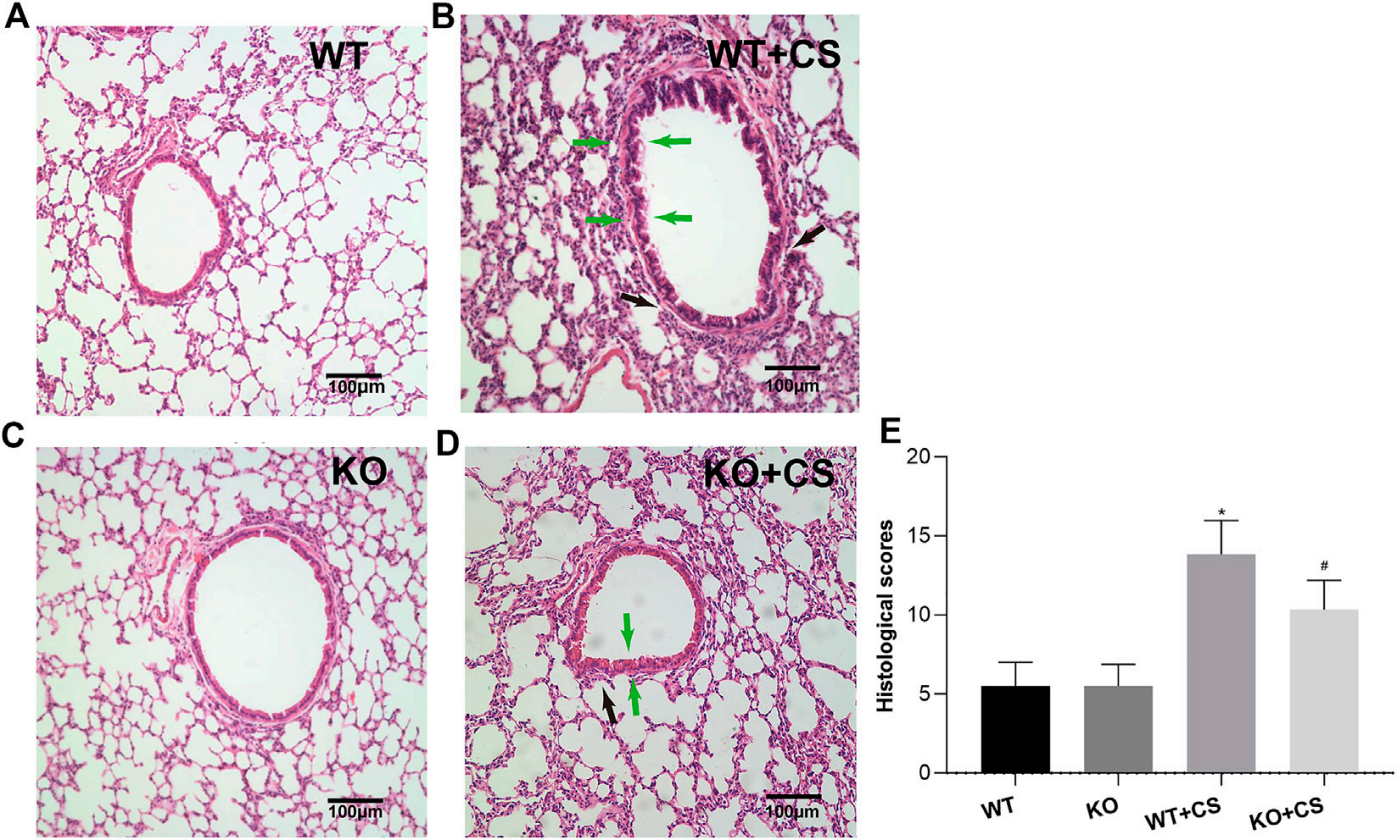
FPR-1 KO reduces CS-induced histological changes in mouse lungs. Representative photomicrographs of HE stained sections of lung tissues from each group were shown. **(A)** WT group, **(B)** WT + CS group, **(C)** KO group, **(D)** KO + CS group, and **(E)** Histological scores. Black arrow: peribronchial inflammatory cells; green arrow: airway epithelium thickening. *p < 0.05 vs. WT group, #*p* < 0.05 vs. WT + CS group. *n* = 6 in each group. Abbreviations: CS, cigarette smoke; WT, wild type; KO, knockout.

**FIGURE 2:**
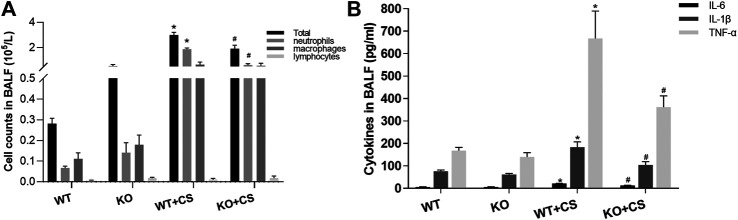
FPR-1 KO diminishes cell counts and pro-inflammatory cytokines in BALF after CS exposure. **(A)** Total cells, neutrophils, macrophages, and lymphocytes in the BALF and **(B)** IL-1β, IL-6, and TNF-α concentrations in the BALF. *p < 0.05 vs. WT group, #*p* < 0.05 vs. WT + CS group. *n* = 6 in each group. Abbreviations: CS, cigarette smoke; WT, wild type; KO, knockout; BALF, bronchoalveolar lavage fluid.

After four-week repeated exposure of mainstream CS, HE staining showed the pathological changes of the mice including the thickening of airway epithelium and peribronchial inflammatory cell infiltration, resulting in a significantly increased overall histological scores (Figure 1). Meanwhile, inflammatory cell counts and the levels of IL-1β, IL-6, and TNF-α increased in the BALF (Figures 2A,B). However, in the KO + CS group, these pathological changes, increased release of inflammatory cytokines, and neutrophils in the BALF were significantly attenuated.

### mRNA Expression Profile and Validation

**FIGURE 3:**
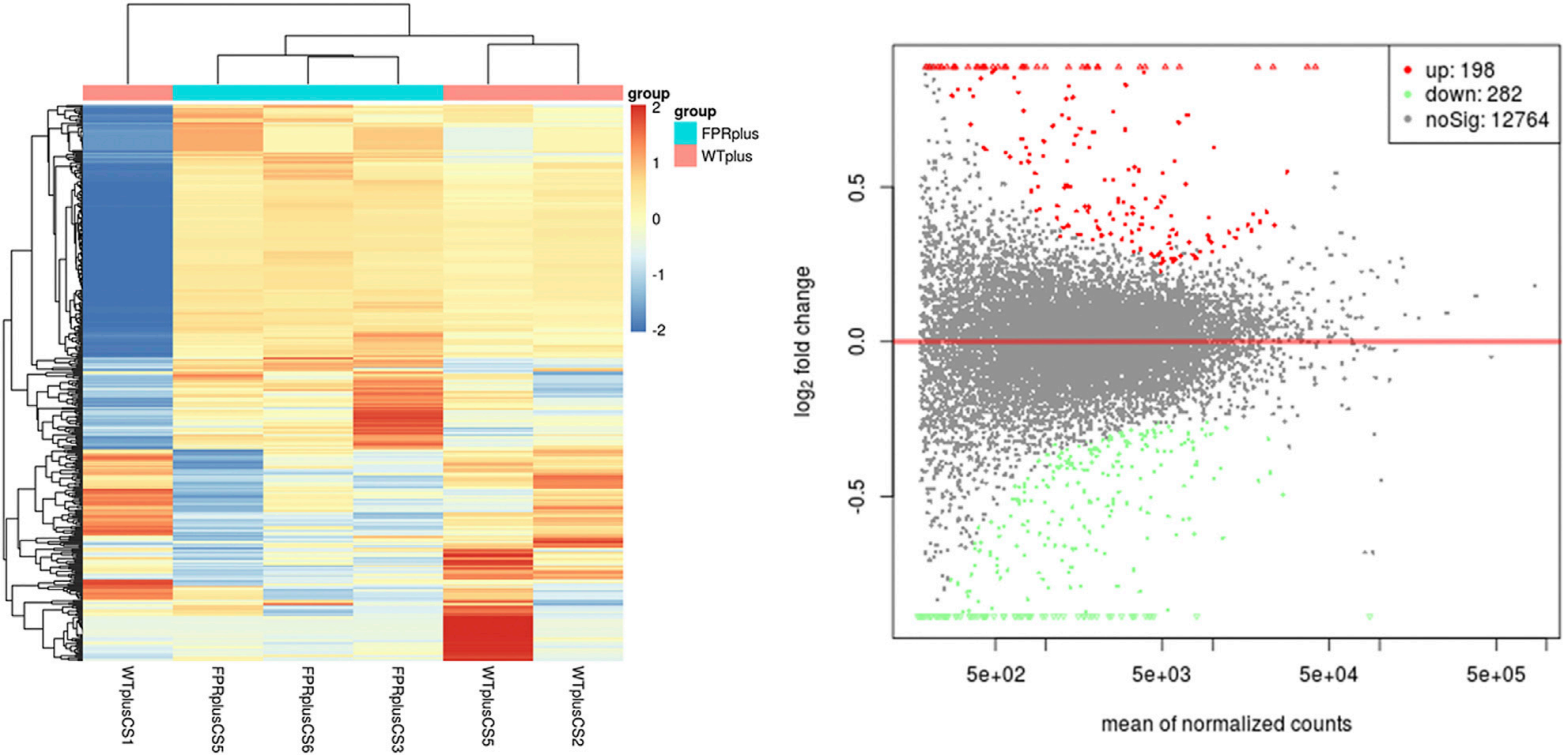
mRNA expression between the WT + CS group and KO + CS group. **(A)** Heat map of mRNAs from mice lung tissues in the WT + CS group and KO + CS group. Each column represents the expression profiles of a mouse lung tissue, and each row corresponds to mRNA. “Red” indicates the higher expression level and “blue” indicates the lower expression level. **(B)** MA plots comparing the expression of mRNAs between the WT + CS group and KO + CS group. The red dots represent up-regulated mRNAs having *p* values < 0.05 in the two groups; the green dots represent down-regulated mRNAs having *p* values < 0.05. Abbreviations: CS, cigarette smoke; WT, wild type; KO, knockout.

**FIGURE 4:**
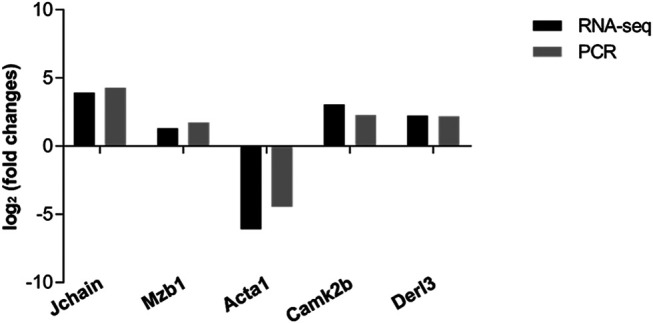
Validation of RNA-sequencing data by qRT-PCR. Five differentially expressed mRNAs were validated by qRT-PCR, including four up-regulated mRNAs and one down-regulated mRNA. The heights of the columns in the chart represent the mean expression value of log2 fold changes (smoke/control). All data were normalized to *β*-actin gene expression.

To further investigate the potential mechanism of FPR-1 in CS-induced airway inflammation, we performed the transcriptomic gene study on lung tissues of the WT + CS and FPR-1 KO + CS mouse. Compared with WT + CS mice, a total of 198 up-regulated genes and 282 down-regulated genes were detected in FPR-1 KO + CS mice (Figure 3). Among these differentiated expressed genes, five genes were selected randomly to validate the sequencing results. Consistent with sequencing results, the expression of Jchain, Mzb1, Camk2b, and Derl3 were increased in FPR-1 KO + CS mice than WT + CS mice, and Acta1 had the opposite trend, suggesting the reliability of the transcriptomic data (Figure 4). The top 20 differentially expressed mRNAs identified by transcriptomic analysis are listed in Table 1.

**TABLE 1:** Detailed information of the top 10 up-regulated and top 10 down-regulated mRNAs.

Gene symbol	Description	Regulation direction	Fold change	*p* value
Jchain	Immunoglobulin joining chain	Up	11.10	9.53E−40
amk2b	Calcium-/calmodulin-dependent protein kinase II, beta	Up	6.04	1.71E−41
Derl3	Der1-like domain family, member 3	Up	4.35	1.55E−08
H2-M2	Histocompatibility 2, M region locus 2	Up	3.98	7.26E−4
Mzb1	Marginal zone B and B1 cell-specific protein 1	Up	3.92	2.46E−26
Prg2	Proteoglycan 2, bone marrow	Up	3.37	1.33E−05
Gm21541	Predicted gene, 21,541	Up	3.20	2.49E−07
Cacna1h	Calcium channel, voltage-dependent, T type, alpha 1H subunit	Up	3.09	1.80E−06
Zfp981	Zinc finger protein 981	Up	3.09	3.22E−09
Mt4	Metallothionein 4	Down	1.41E−3	o
Hrnr	Hornerin	Down	2.26E−3	1.36E−29
Serpinb3b	Serine peptidase inhibitor, clade B, member 3 B	Down	2.31E−3	1.53E−77
Myh1	Myosin, heavy polypeptide 1, skeletal muscle, adult	Down	6.03E−3	1.53E−37
Lgals7	Lectin, galactose binding, soluble 7	Down	6.34E−3	1.71E−150
Acta1	Actin, alpha 1, skeletal muscle	Down	1.06E−2	4.29E−42
Lor	Loricrin	Down	1.19E−2	3.09E−228
Rptn	Repetin	Down	1.56E−2	6.23E−116
Tnnc2	Troponin C2, fast	Down	1.83E−2	5.21E−65
Tgm3	Transglutaminase 3, E polypeptide	Down	2.38E−2	2.24E−62

### Functional Enrichment Analysis of DEGs

**FIGURE 5:**
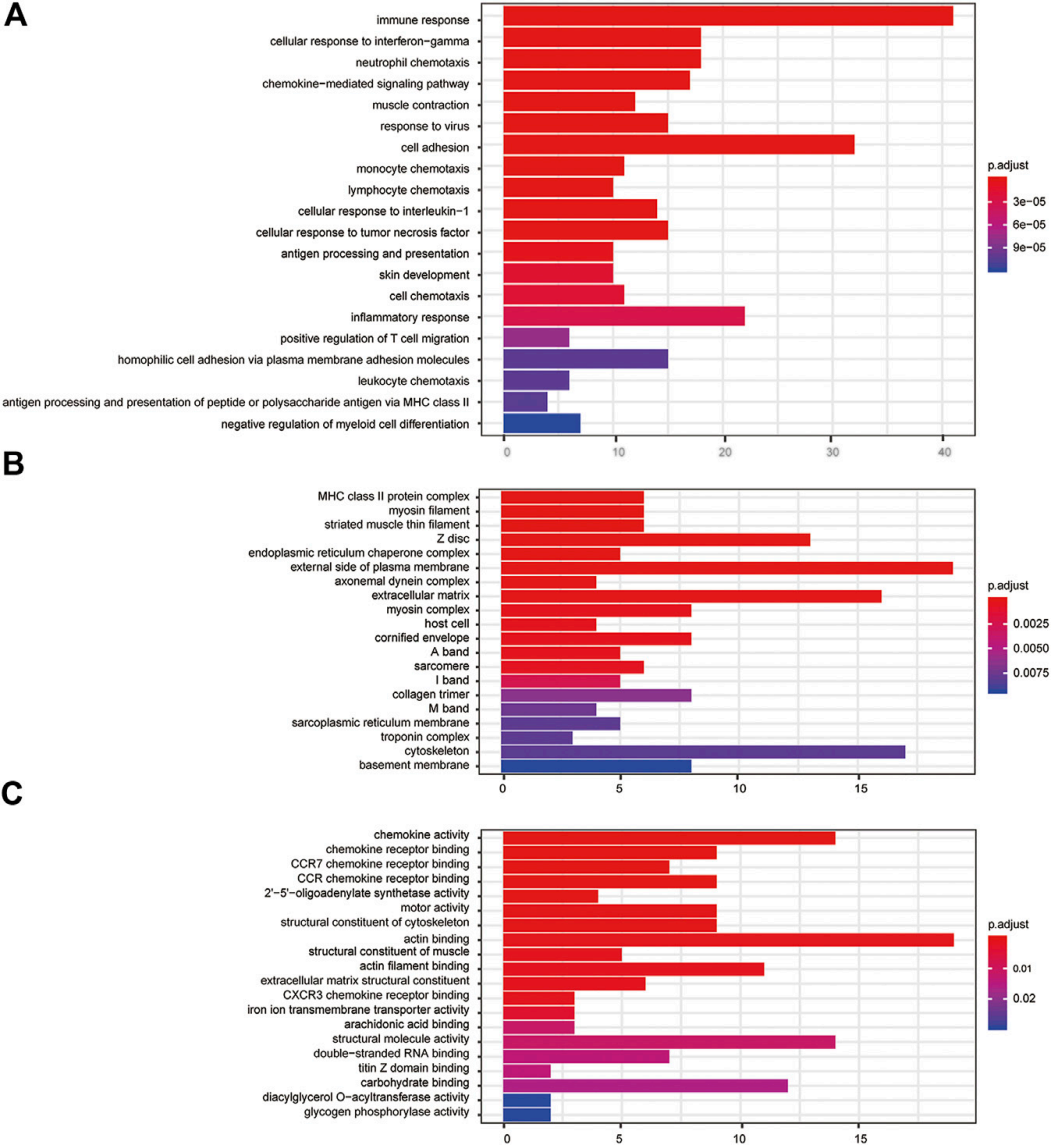
GO analysis. **(A)** Barplots of the biological process enrichment result, **(B)** barplots of the molecular function enrichment result, and **(C)** barplots of the cellular component enrichment result. The colored entries are the significant aggregation (*p* < 0.05), and *p* values are shown on the right side. The lower the *p* value is, the more significant the enrichment is. The length of the bar means the number of genes in the significant aggregation.

**FIGURE 6:**
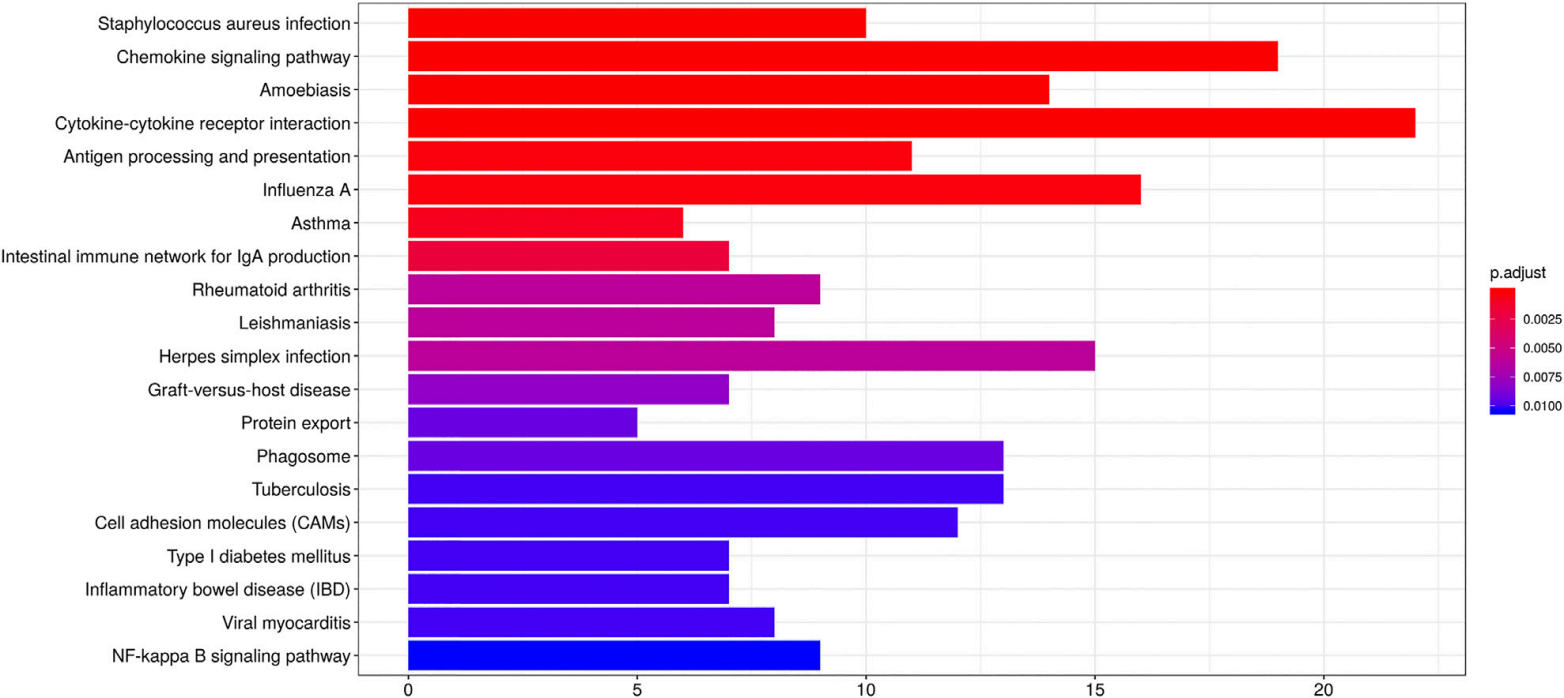
KEGG analysis. Barplots of the pathway enrichment result. The colored entries are the significant aggregation (*p* < 0.05), and *p* values are shown on the right side. The lower the *p* value is, the more significant the enrichment is. The length of the bar means the number of genes in the significant aggregation.

GO analysis suggested FPR-1 KO was relevant to biological process of immune, chemotaxis, inflammatory response, etc., cellular components of MHC class II protein complex, myosin filament, striated muscle thin filament, etc., and molecular function of chemokine activity, chemokine receptor binding, etc. (Figure 5). KEGG pathway analysis further revealed alterations in many pathways including chemokine signaling pathway, cytokine−cytokine receptor interaction and NF-κB signaling pathway, etc. (Figure 6).

### Effects of the Absence of FPR-1 on NF-κB Signaling Pathway

**FIGURE 7:**
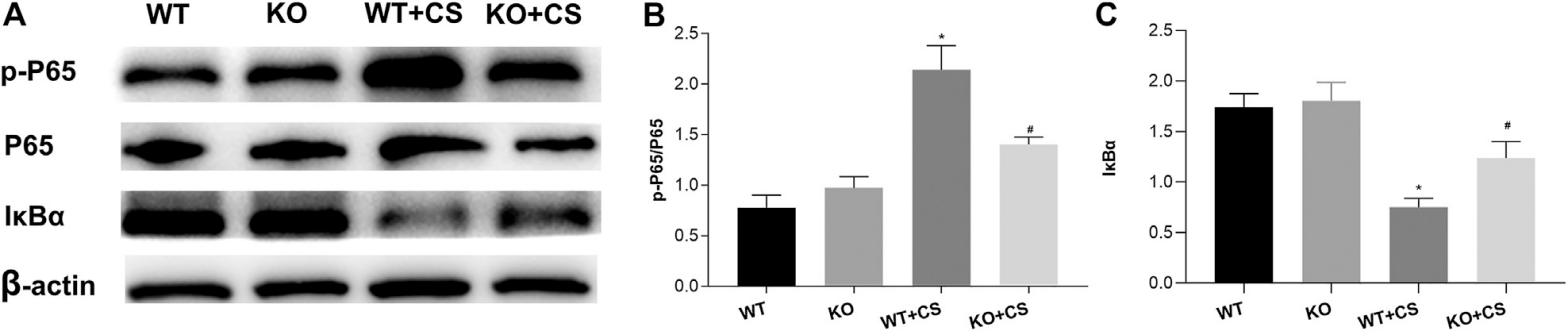
Effect of FPR-1 gene deletion on the NF-κB signaling pathway. **(A)** Effect of FPR-1 gene deletion on IκBα, NF-κB P65, and phospho-NF-κB P65. Tissue samples from KO + CS mice exhibited less NF-κB P65 phosphorylation. **(B)** p-P65/P65 levels were remarkably decreased, and IκBα/β-actin levels were significantly increased after 4-weeks CS exposure in the KO + CS group. *p < 0.05 vs. WT group, #*p* < 0.05 vs. WT + CS group. *n* = 3 in each group. Abbreviations: CS, cigarette smoke; WT, wild type; KO, knockout; P65, NF-κB P65; p-P65, phospho- NF-κB P65.

In order to investigate the underlying mechanism whereby FPR-1 gene deletion could attenuate CS-induced airway inflammation in COPD, based on above KEGG analysis, we examined the expression and phosphorylation of NF-κB p65 in lung tissues collected from WT + CS and KO + CS mice. Compared to the WT + CS group, Western blot analysis indicated a reduction of phospho-NF-κB p65 and an increase of IκBα in the KO + CS group (Figure 7).

## Discussion

In this study, KO of FPR-1 demonstrated a protective effect of airway inflammation induced by CS in a mouse model. To determine the detailed mechanism under FPR-1 modulation, further transcriptional study together with GO and KEGG analysis suggested FPR-1 KO related differentiated expressed genes significantly related to the immune, chemotaxis, and inflammatory response in lungs via cross-talking with a complicated network of signaling pathways. Furthermore, the NF-κB signaling pathway was significantly downregulated in the KO + CS group validated by Western blot analysis, suggesting the absence of FPR-1 may render mice significantly less susceptible to the development of the CS-induced airway inflammation compared to the WT group via the NF-κB signaling pathway.

Cigarette smoking was a crucial risk factor of COPD cases (Barnes et al., 2003). For animal models, smoke exposure is an important simulation of an inflammatory response in airways and lung tissues (John et al., 2014; Wood et al., 2014). Recently, growing studies observed a potential association between FPR- and CS-related diseases and other respiratory disorders, it has been demonstrated that FPR-2 was particularly relevant to neutrophilic inflammation in COPD as the complex milieu of exogenous and host-derived mediators (Bozinovski et al., 2013; Bozinovski et al., 2014). Moreover, recent studies revealed FPR-1 played a critical role in regulating lung inflammation and injuries in bronchopulmonary dysplasia and it also has been demonstrated that FPR-1 can reduce lung injury by mediating neutrophil recruitment in endotoxin-induced ALI (Grommes et al., 2014; Kim et al., 2018). Our group also observed that pro-inflammatory responses in the alveolar epithelium can be related with the FPR-1 and NF-κB signaling pathway in response to mitochondrial damage-associated molecular patterns (MTDs) stimulation (Zhang et al., 2018). Besides, studies showed that the FPR-1 antagonist, such as propofol, may benefit critically ill septic patients through alleviating alveolar wall disruption, edematous changes, and neutrophil infiltration (Chen et al., 2018). Furthermore, FPR-1 antagonist cyclosporin H reduced neutrophil recruitment in acid-induced lung inflammation (Dorward et al., 2017), and formyl peptides have been reported to be present in tobacco leaves and FPRs promoted the progression of COPD in smokers (De Cunto et al., 2020a) ,which further elicited FPR-1 inhibition might provide a novel insight into inflammation control in COPD. In this study, FPR-1 knockout can protect mice against from CS-induced airway inflammation. Furthermore, it has demonstrated that modulation of FPR signaling with antagonists mitigates pulmonary inflammation in mice after smoking cessation (De Cunto et al., 2018). In general, the FPR-1 signaling pathway may have a function on treatment and prevention of CS-induced airway inflammation and relate respiratory disorders.

Although previous studies have reported that the relationship between FPR-1 and smoking-induced lung emphysema in mice, the mechanism under FPR-1 modulation has not been fully examined (Cardini et al., 2012). To further investigate the potential mechanism of FPR-1 mediated airway inflammation induced by CS, a transcriptomic gene study, and related bioinformatics analysis were performed. In our GO process, FPR-1 gene knockout had multiple effects on many biological processes, including not only an immune response but also neutrophil chemotaxis and chemokine-mediated signaling pathway. In the present research, cigarette smoke can recruit neutrophils and inflammatory monocytes to the lungs through activation of innate immune cells such as macrophages and airway epithelial cells to release multiple chemotactic factors (Barnes, 2016). The chemokine networks played a key role in the pathogenesis of COPD. It has been reported that C-X-C motif chemokine 5 (CXCL5) might coordinate with the granulocyte colony–stimulating factor (G-CSF) in the pathogenesis of neutrophilic inflammation in COPD (Chen et al., 2019). Substantially, the direct inhibition of neutrophil migration by using roflumilast can improve forced expiratory volume in 1 s (FEV_1_) and reducing exacerbation frequency in COPD patients(Wedzicha et al., 2016; Dunne et al., 2019). Previous studies have suggested that FPR-1 signaling can mediate neutrophils and macrophages responses to inflammation, and it is well known that FPR-1 is one of the most extensively studied receptors involved in neutrophil chemotaxis (Ye et al., 2009; Dahlgren et al., 2016). In mice exposed to cigarette smoking, the recruitment of neutrophils by CSE stimuli on FPR was necessary to develop airway remodeling and pulmonary emphysema in the COPD murine model (Cardini et al., 2012). This study indicated that FPR-1 gene knockout may play a crucial role in many biological processes, molecular functions, and cellular components in CS-induced immune, chemotaxis, and inflammatory response.

In KEGG pathway analysis, this study found that in which a complicated network of signaling pathways, including not only toll-like receptors, but also other chemokine signaling pathways involving immune-inflammatory responses. The inflammatory immune response has been implicated in the pathogenesis of COPD. Cigarette smoking elicits the inflammatory response through the chemokine signal pathway to recruit neutrophils and other inflammatory cells to the lungs. Noticeably, the NF-κB signaling pathway was associated with the FPR-1 gene with dominant significance in the KEGG pathway analysis. In this study, Western blot analysis demonstrated the decreased activation of the NF-κB signaling pathway in FPR-1 knockout mice after four-week smoke exposure. As we know, the NF-κB signaling pathway is important in the immune and inflammatory response and modulates cell proliferation, apoptosis, adhesion, invasion, and angiogenesis in cells (Zhang et al., 2017). And NF-κB activation has been reported during lung inflammation induced by subchronic cigarette smoke exposure in mice, which was observed in the present study (Vlahos et al., 2006). FPR-1 gene deletion has been reported to take protective effects via the downregulation of the NF-κB pathway in surgically induced endometriosis (Fusco et al., 2018). And in the colitis model of mice, it has been indicated that there was reduced NF-κB translocation into the nucleus in absence of the FPR-1 gene (Di Paola et al., 2019). Taken together, the protective effects of the FPR-1 gene deletion in CS-induced airway inflammation may depend on the downregulation of the NF-κB pathway. Collectively, our data show that the mouse model with absence of FPR-1 gene displayed reduced inflammation after 4-week CS exposure, which may in part through the regulation of NF-κB, suggesting it as a new target to control the CS-induced airway inflammation in COPD.

There were some limitations in this study that should be considered. First, this study is a primary basic study *in vivo*, and it would be more convincing that mice were exposed to long term of CS to explore the role of FPR-1 in the pathophysiology of COPD. Meanwhile, its translation to a clinical study needs a long way. Second, due to limited time and research space, the sample size is needed to expand to increase the statistic power and get more convincing conclusions. Because of limited funding, different strains of mice were not used in the present study, which may influence the clinical phenotypes after CS exposure (De Cunto et al., 2020b). And for the purpose of avoiding pregnancy during the experiment, the CS exposure experiment was not performed in FPR-1 knockout female mice. Third, more validation work including studies *in vitro* and preclinical studies is needed to explore greater mechanistic insight in the future.

In summary, our study reveals that knockout of FPR-1 could attenuate CS-induced airway inflammation, possibly via its related immune, chemotaxis, and inflammatory responses and inhibition of NF-κB activation. Targeting this pathway may help to diminish CS-induced airway inflammation and is thus a potential therapeutic option to develop new therapies for CS-induced airway disorders.

## Data Availability

Raw transcriptomic data were uploaded in China National Center for Bioinformation-National Genomics Data Center (accession: CRA003586).
