# Supplementary material for: Knockout of Formyl Peptide Receptor-1 Attenuates Cigarette Smoke–Induced Airway Inflammation in Mice
Source: Front Pharmacol. 2021 Apr 26;12:632225. doi: 10.3389/fphar.2021.632225 (PMC8110203; doi:10.3389/fphar.2021.632225)
Supplement: Supplementary file 1 [file Table1.DOC]

**Supplementary Table 1: The primer sequences** information table

| **Primer name** | **Sequence (3'-5')** |
| --- | --- |
|  |  |
| Jchain | F: TTGTAACAGGTGACGACGAAG |
|  | R: GGAAGGGATGATCCTAGAGGTA |
| Camk2b | F: CAGACCCTAATGAAGATGGAGA |
|  | R: GCTGAGTGGACAAACACCCTA |
| Derl3 | F: GTCTCAGGTGCAGAGGACAAG |
|  | R: CTACAAGGCAAATGGAAGGAA |
| Mzb1 | F: TGGATTGCAGGAGCTGAGTG |
|  | R: GAGACGCTTCATCTGGTTCA |
| Acta1 | F: GTGCGACGAAGACGAGAC |
|  | R: CCACGATGGATGGGAACA |
| β-actin | F: CAGGTCATCACTATTGGCAAC |
|  | R: TCTTTACGGATGTCAACGTCA |
